# Identifying In-*Trans* Process Associated Genes in Breast Cancer by Integrated Analysis of Copy Number and Expression Data

**DOI:** 10.1371/journal.pone.0053014

**Published:** 2013-01-30

**Authors:** Miriam Ragle Aure, Israel Steinfeld, Lars Oliver Baumbusch, Knut Liestøl, Doron Lipson, Sandra Nyberg, Bjørn Naume, Kristine Kleivi Sahlberg, Vessela N. Kristensen, Anne-Lise Børresen-Dale, Ole Christian Lingjærde, Zohar Yakhini

**Affiliations:** 1 Department of Genetics, Institute for Cancer Research, Oslo University Hospital Radiumhospitalet, Oslo, Norway; 2 K. G. Jebsen Centre for Breast Cancer Research, Institute for Clinical Medicine, University of Oslo, Oslo, Norway; 3 Laboratory of Computational Biology, Computer Science Department, Israel Institute of Technology, Haifa, Israel; 4 Biomedical Informatics Lab, Department of Computer Science, University of Oslo, Oslo, Norway; 5 Centre for Cancer Biomedicine, University of Oslo, Oslo, Norway; 6 Division of Cancer Medicine and Radiotherapy, Department of Oncology, Oslo University Hospital Radiumhospitalet, Oslo, Norway; 7 Institute for Clinical Epidemiology and Molecular Biology (EpiGen) Akershus University Hospital, Akershus, Norway; 8 Agilent Laboratories, Tel Aviv, Israel; University of Turin, Italy

## Abstract

Genomic copy number alterations are common in cancer. Finding the genes causally implicated in oncogenesis is challenging because the gain or loss of a chromosomal region may affect a few key driver genes and many passengers. Integrative analyses have opened new vistas for addressing this issue. One approach is to identify genes with frequent copy number alterations and corresponding changes in expression. Several methods also analyse effects of transcriptional changes on known pathways. Here, we propose a method that analyses in-*cis* correlated genes for evidence of in-*trans* association to biological processes, with no bias towards processes of a particular type or function. The method aims to identify *cis*-regulated genes for which the expression correlation to other genes provides further evidence of a network-perturbing role in cancer. The proposed unsupervised approach involves a sequence of statistical tests to systematically narrow down the list of relevant genes, based on integrative analysis of copy number and gene expression data. A novel adjustment method handles confounding effects of co-occurring copy number aberrations, potentially a large source of false positives in such studies. Applying the method to whole-genome copy number and expression data from 100 primary breast carcinomas, 6373 genes were identified as commonly aberrant, 578 were highly in-*cis* correlated, and 56 were in addition associated in-*trans* to biological processes. Among these *in-trans process associated and cis-correlated* (iPAC) genes, 28% have previously been reported as breast cancer associated, and 64% as cancer associated. By combining statistical evidence from three separate subanalyses that focus respectively on copy number, gene expression and the combination of the two, the proposed method identifies several known and novel cancer driver candidates. Validation in an independent data set supports the conclusion that the method identifies genes implicated in cancer.

## Introduction

Genomic copy number alterations resulting from genomic instability are commonly observed in cancer [1], [2]. Substantial effort has been invested in identifying aberration events playing a critical role in the disease development. In breast carcinomas, the genomic architectural changes are diverse and involve various events such as loss and gain of whole chromosome arms, inversions, translocations, and more focal gains and losses [3], [4]. Several array comparative genomic hybridization (aCGH) studies of breast tumors and breast cancer cell lines point to commonly observed gains and losses on regions of chromosome 8, 13 and 17 – regions known to contain breast cancer associated genes such as *BRCA2*, *ERBB2* and *MYC* [5], [6], [7], [8], [9].

Recurring aberrations in tumors may be indications of selection driven by changes in the expression of key genes in the affected regions. Since recurrent segmental gains and losses frequently involve several genes, their relative contribution to increased or decreased cell viability and proliferation cannot be inferred from copy number alone. This problem, often portrayed as distinguishing between ‘drivers’ and ‘passengers’, is a key challenge in the task of linking copy number alterations to genes and processes involved in cancer development and progression. One way to proceed would be to focus on genes for which copy number variation substantially affects gene expression. Integrated analyses of copy number and gene expression data have revealed that the strength of the in-*cis* correlation between copy number and expression varies extensively between genes [10], and subsets of genes with high correlation have been identified and proposed as candidate driver genes [10], [11], [12], [13], [14], [15].

It has been suggested that the oncogenic effect of molecular alterations is to cause perturbations at the network level, leading cells to malignant phenotypic states (see, e.g. [16]). Several studies have aimed at identifying pathways and networks perturbed by copy number aberrations, thus establishing associations between genomic profiles and aberrant pathways in cancer [17], [18], [19], clinical outcome and survival [13], [20], [21], [22], [23]. One may ask whether a particular gene through its genomic aberrations has an effect on higher-order phenotypes such as processes, pathways and networks. A natural way to approach this would be to first investigate how other genes are affected by the aberration, and second to study whether any biological processes are overrepresented in the list of affected genes. Following this idea, we propose a workflow for integration of copy number and gene expression data based on the stepwise application of a series of gene selection criteria. The method combines correlation analysis, regression analysis, and gene set enrichment, and to avoid confounding effects, the method adjusts for co-occurring copy number aberrations. A key element of the approach is the direct integration of a statistical enrichment step enabling the assignment of statistical confidence to in-*trans* associations between genes and biological processes. The resulting genes are referred to as *in-trans process associated and cis-correlated* (iPAC) genes.

The purpose of combining in-*cis* and in-*trans* analyses is here to identify genes that are *cis*-regulated and for which the correlation structure in the gene expression data provides further support for a role in the alteration of cell phenotype in cancer. The method was applied to a matched data set of aCGH and mRNA expression from 100 well-characterized human primary breast tumors [24], [25], [26], [27], and subsequent application to a second, independent breast cancer cohort showed consistent behavior of the iPAC genes found in the first data set. A small selection of iPAC genes were further studied using siRNA knockdown experiments.

## Materials and Methods

### Ethics statement

The study was approved by the Norwegian regional committee for medical research ethics, Health region II (reference number S-97103), and patients have given written consent for the use of material for research purposes.

### Patient samples and array experiments

Primary breast carcinoma samples from 100 patients previously described as part of the MicMa cohort were used [24]. All samples were fresh frozen and contained at least 40% tumor cells. The majority of the tumor specimens represent tumor size T1/T2, node status N0/N1 (9/11), and histological grade 2 or 3. Tumor DNA was extracted using an ABI 341 Nucleic Acid Purification System (Applied Biosystems, CA, USA) according to the manufacturer's protocol. Tumor RNA was isolated using TRIzol reagent (Invitrogen, CA, USA) as previously described [28]. The subtype classification deriving from mRNA expression has previously been presented [29]. The aCGH and the mRNA expression data sets have previously been published [26], [30]. The expression data (measured using Agilent 4 by 44K one-color oligonucleotide arrays) are available in Gene Expression Omnibus (GEO) with accession number GSE19783 [26], and the copy number data (measured using Illumina Human-1 109K BeadChip SNP arrays) are available on request to OCL. A breast cancer data set from the University of North Carolina, Chapel Hill (UNC), including 73 samples profiled on the same array platforms as described above, was used for validation (see [25], [31] for details). Genomic locus annotation for all analyzed data is based on the human genome build hg17.

### Overview of analysis

**Figure 1:**
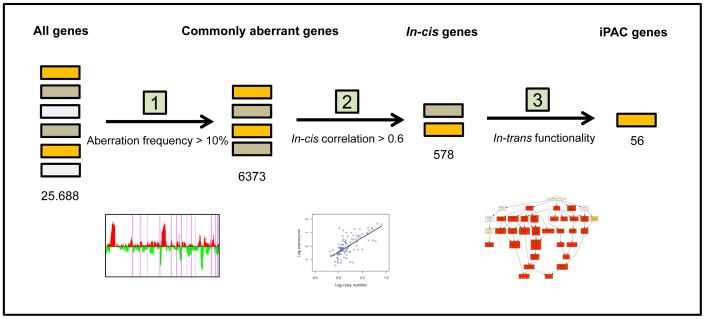
Workflow of the proposed method to identify iPAC genes. (1) Starting with all genes, the commonly aberrant genes are selected as those that have more than 10% gains or losses; (2) Next, those genes which in addition have an in-*cis* Pearson correlation above 0.6 are selected and referred to as in-*cis* genes; (3) Finally, statistical enrichment analysis is performed to assess in-*trans* functionality, leading to identification of the 56 iPAC genes.

The proposed method is based on the stepwise application of a series of gene selection criteria, and a core element is the use of a rigorous statistical enrichment technique to reveal significant associations in *trans* between the selected genes and biological processes (see Figure 1). This enrichment step is combined with a novel correction method designed to alleviate the problem of co-occurring copy number alterations across the genome.

### Segmentation

Copy number data were log2-transformed, and each sample was segmented by fitting a piecewise constant regression function to the data using the piecewise constant fitting (PCF) algorithm in the R package *copynumber* [3], [32], [33], [34]. A fitted value (“PCF value”) was then obtained for each segment (and was inherited by each probe in the segment) by averaging the log-transformed copy number values for all probes located in that segment. The user controls the trade-off between sensitivity and specificity with a penalty parameter (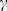) and the minimal number of probes per segment (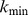). We chose 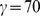 which is fairly conservative and thus provides robustness against the presence of potential local (spurious) trends which are common in aCGH data due to varying GC-content and other reasons (see [34] for details), and use the default value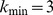.

### Matching copy number and expression values

In order to obtain matching copy number and expression data sets, we first identified all expression probes annotated with a gene symbol in the data set. For each such probe, the copy number probe mapping to the nearest location in the genome was identified. Copy number and gene expression data were then averaged over the corresponding probe values for each gene symbol, resulting in a unique copy number value and expression value for each patient and each gene. The corresponding pair of values was assigned a genomic position by averaging over the locations of the expression probes associated with the gene symbol. Analogous methods are also used in other studies [12], [35], [36]. This procedure yielded two 25,688×100 matrices of copy numbers and corresponding gene expressions, where each row represents a gene and each column a patient sample.

### Aberration calling

To call aberrations, a parameter 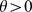 determining the sensitivity of the aberration calling (and hence what is considered a significant aberration) was introduced. Probes with a PCF value larger than 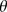 were called as gains, and probes with a PCF value less than 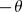 were called as losses. Following the recommended practice for threshold selection in the R package *copynumber* [34], we concluded that 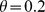 was an appropriate threshold, which is very similar to the threshold used in [33] where a subset of the copy number data considered in this paper was analyzed.

### Identification of common gains and losses

To identify genomic loci where the copy number events are skewed towards either gain or loss, a sign test was applied. Let 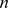 denote the total number of samples with an aberration in a particular locus, and suppose 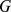 of these aberrations are gains and 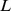 are losses (so that 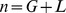). Modeling the number of gains as a binomial distribution with 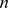 draws and success probability 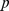, 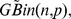 we may formally infer whether gains are overrepresented by testing the null hypothesis 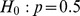 against the alternative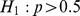. Using the difference 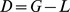 as the test statistic, we want to determine the rejection region 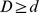 where 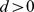 is a given threshold. Since 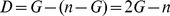, we have that
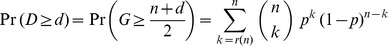
where 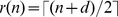. Assuming a significance level of 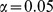, we seek the least integer 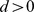 for which we have 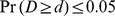 for all 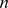 under the null hypothesis of 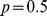. In practice (see Results) the number of aberrations never exceeds 38 in any given locus, and 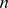 may be restrained correspondingly above. A simple calculation then shows that the appropriate threshold is 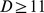, and this value was used in the analyses. Thus, all genes for which 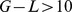 were defined as being commonly gained.

By an analogous argument, all genes for which 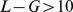 were defined as being commonly lost. Whenever 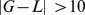, the gene was referred to as being commonly aberrant. Note that the purpose of this step was to filter out the bulk of aberrant genes with no indication of skewness towards either gain or loss, and hence the above significance criterion was designed to be very mild and did not take into account multiple comparisons.

### Identification of in-*cis* correlated genes

To seek the genes for which the expression is significantly influenced by the copy number, we identified in-*cis* correlated genes. To identify significant in-*cis* correlations between log copy number and log gene expression, the in-*cis* correlations of all the commonly aberrant genes were compared to a background distribution of in-*cis* correlations. The background distribution was generated by performing 2000 shuffling simulations where in each, only the gene order in the aCGH data set was shuffled and the in-*cis* correlations were recalculated. By selecting the genes with (Pearson) in-*cis* correlation 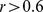 we achieved a false discovery rate (FDR) of less than 2%. This cut-off corresponds to a coefficient of determination of 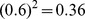, meaning that at least 36% of the variation in log-expression is accounted for by the in-*cis* variation of log-copy number.

The above procedure corresponds to keeping only the genes 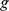 for which the following log-linear model provides a good fit to the observed copy number and gene expression levels:
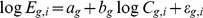
(1)where 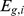 and 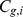 denote respectively expression and copy number of gene 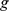 in the *i*th sample, and 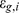 are independent and identically distributed noise terms. Equation (1) implies that log expression is a linear function of log copy number (and noise). Suppressing the gene subscript and ignoring the noise term, equation (1) is equivalent to 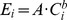 (where 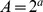, assuming logarithms in base 2). Accordingly, model (1) is flexible enough to allow both linear (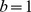) and nonlinear (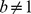) relations between the expression and the copy number of a gene.

### In-*trans* correlation analysis

The purpose of this step was to quantify the level of association between the in-*cis* genes and other genes. To do this, we considered the correlation between the in-*cis* gene and all other genes. A potential problem in this context is that genes close to each other on the same chromosome may be affected by the same copy number alterations, with inflated correlation between the expression levels of the two genes as a possible result. Thus, co-occurring copy number aberrations can act as a confounding factor, and this should be taken into account when assessing potential expression-mediated effects of one gene on another. To avoid this problem we calculated for each gene 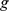 the residual log expression values 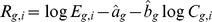 over all the samples, where the coefficients 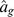 and 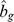 were found by fitting the model in (1), and quantified the in-*trans* effect of an in-*cis* gene 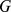 on gene 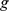 by the Pearson correlation between the observed log expressions 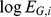 of the in-*cis* gene and the residual log expressions 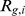 of the gene in *trans*.

### Identification of in-*cis* genes associated in-*trans* with processes

In order to identify in-*cis* genes that were associated with processes in *trans*, we considered 8284 gene sets defined by Gene Ontology (GO) biological process terms [37]. Using each in-*cis* gene 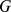 in turn as a pivot, all other genes 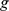 were ranked according to the correlation between 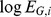 and 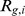
(from high positive correlation to high negative correlation), and an enrichment score was calculated for each GO term in the ranked list of genes. This was done separately for the genes in the top and the bottom of the ranked list. The enrichment score was defined as the p-value from the minimum hypergeometric (mHG) test (see [38], [39] for details). Such scores were calculated for each in-*cis* gene and each GO biological process term. For further analysis, we only considered associations between in-*cis* genes and GO terms with a p-value score p<0.05 (after Bonferroni correction).

To obtain empirical p-values for the associations selected above, 100 random simulations were performed. In each simulation instance, we shuffled the order of the samples in the residual expression data set only and recalculated all enrichment scores. This approach preserves existing expression dependencies between genes. Let 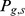 be the enrichment score (mHG p-value) of the association between in-*cis* gene 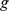 and gene set (GO term) *s*, and let 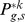 be the enrichment score of this association in the *k^th^* simulation instance (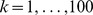). We considered an in-*cis* gene 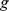 to be significantly associated with a gene set *s* if 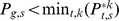, where 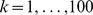 and *t* ranges over all in-*cis* genes. That is, a relation between an in-*cis* gene and a gene set was called significant if the observed enrichment score (mHG p-value) was less than the enrichment scores obtained for that gene set for all in-*cis* genes in all the simulations. This step alleviates differences in attainable p-values due to correlated null hypotheses (the GO gene sets have strong overlaps and genes within a set may be strongly dependent).

### Enrichment analysis using GOrilla

GOrilla (http://cbl-gorilla.cs.technion.ac.il/) [38], [39] was used with default parameters to investigate and visualize the enrichment of GO biological process in ranked lists of selected gene sets.

## Results

We have presented a computational framework for identification of aberrant genes potentially leading to a substantial shift in transcriptional programs. The proposed method was applied to matched copy number and expression data from a cohort of 100 breast carcinomas. The resulting iPAC genes were further validated in a data set from another breast cancer cohort. The workflow of our approach is depicted in Figure 1 and details are provided in Materials and Methods.

### Common aberrations

**Figure 2:**
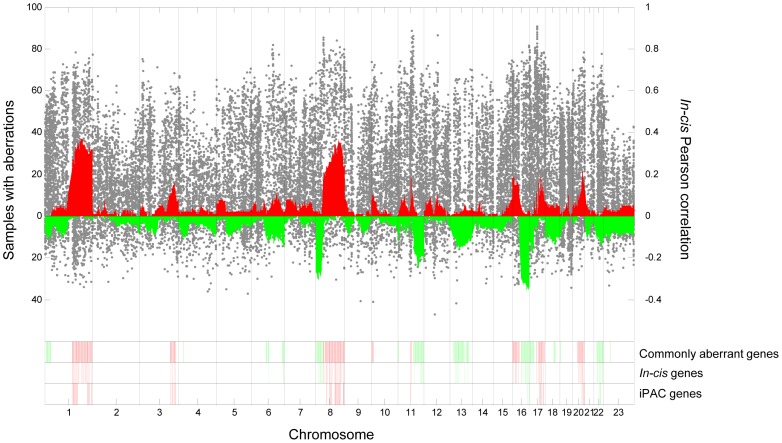
Copy number aberrations and in-*cis* correlations. The frequency of samples with gains (red) and losses (green) is shown at the top. Each gray point shows the level of in-*cis* correlation between copy number and expression for a particular gene. The chromosomal positions of the genes selected in our workflow are shown at the bottom. This includes commonly aberrant genes (n = 6373; upper band), in-*cis* genes (n = 578; middle band), and the iPAC genes (n = 56; lower band). Colors indicate whether the gene is most frequently amplified (red) or deleted (green).

The first step of our workflow was the identification of genes that were commonly aberrant between the patient samples. Among the 25,688 genes profiled, a total of 6373 genes were found to be commonly aberrant, of which 3499 were commonly amplified and 2874 commonly deleted (notice that by the definition of commonly aberrant genes given in Materials and Methods, a gene cannot be both commonly amplified and commonly deleted). These genes are scattered throughout the genome with highest frequency on chromosomes 1, 8, 11, 13, 16, and 17 (Figure 2; Figure S1A). For all genes combined, 7.5% of the variance of the expression values was explained by copy number alterations in *cis*. Considering only commonly aberrant genes, this fraction increased to 11.5%.

### In-*cis* associations

**Figure 3:**
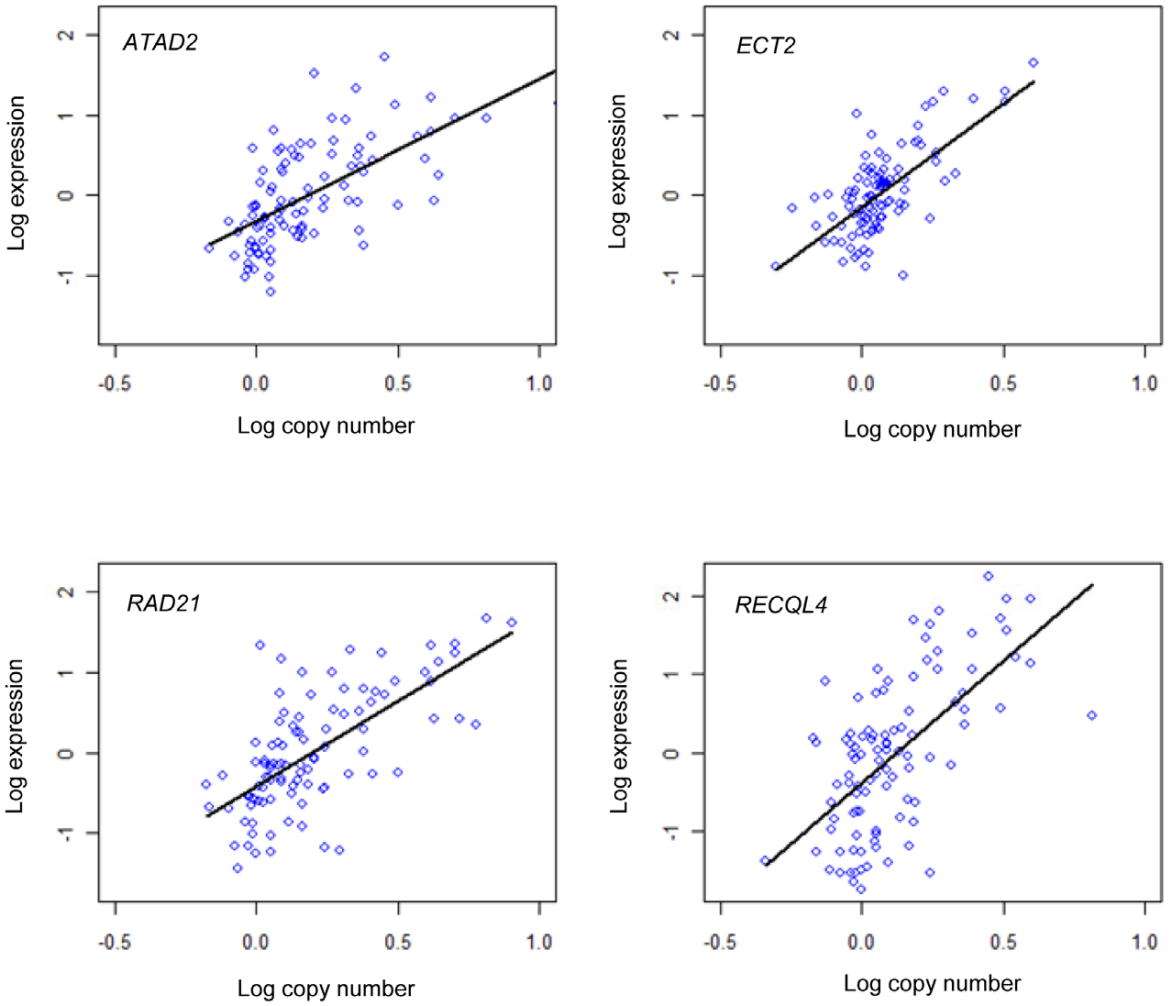
Association between expression and copy number. Linear regression of log-expression as a function of log-copy number for four selected iPAC genes.

The in-*cis* correlation is shown for all genes in Figure 2 and Figure S1B. Ranking the 6373 commonly aberrant genes according to their in-*cis* correlation reveals that the genes with highest correlation are enriched with the GO terms of DNA repair, cell cycle, DNA recombination, and chromatin modification and organization (see Figure S2 and Table S1 for a full list of results). Genes with high in-*cis* correlation (Pearson's r>0.6) were selected among the commonly aberrant genes, resulting in 578 in-*cis* genes (see Figure 2 and 3, and Table S2). These genes were predominantly found on chromosomes 1, 8, 16, and 17. Of these, 423 genes were commonly amplified and 155 commonly deleted (Figure 2).

The in-*cis* genes included known cancer-associated genes such as *ERBB2*, *MAP3K7*, *MDM4*, *FGFR1*, *CCND1* and *FADD*. Further annotation of the 578 genes showed that 19% code for enzymes, 8% regulators of transcription, 7% transporters, 4% kinases, 2% peptidases, and 2% phosphatases (Figure S3A). The remaining genes encode various sorts of proteins, e.g. zinc finger proteins, ribosomal proteins, RNA binding proteins, and mitochondrial proteins (see Table S2 for description). The fraction of the variance in expression explained by copy number alterations increased to 46.6% when considering only the in-*cis* genes. Although the in-*cis* genes exhibit strong correlation between copy number and expression, a substantial proportion of the variability in these genes across samples is also related to other influences. Thus, their expression reflects copy number as well as various other factors.

### In-*trans* associations to biological processes

**Figure 4:**
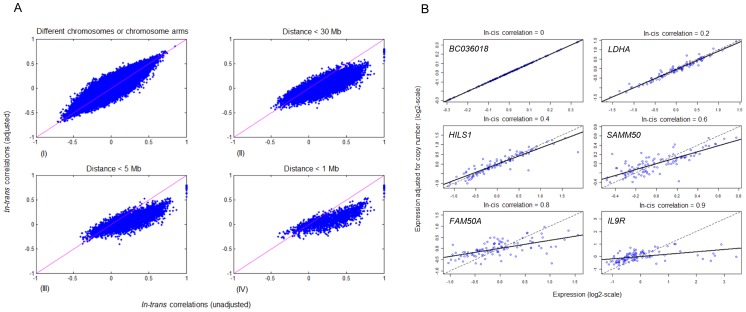
Effect of using copy number-adjusted residual expression. (**A**) Comparison of in-*trans* correlations calculated with and without adjustment for in-*cis* correlation, i.e. copy number-adjusted-residual expression. In each panel, the x-axis represents the in-*trans* correlation without adjustment for in-*cis* correlation, and the y-axis represents the in-*trans* correlation with adjustment for in-*cis* correlation. The diagonal lines extend from (−1,−1) to (1, 1). Each point represents one pair of genes among all the 578×25,688 gene pairs (*G*, *g*) where *G* is an in-*cis* gene and *g* denotes any gene; (I) All pairs for which *G* and *g* are either on different chromosomes or on the same chromosome but on different arms; (II) All pairs for which *G* and *g* are within a distance of 30 Mb from each other; (III) All pairs for which *G* and *g* are within a distance of 5 Mb from each other; (IV) All pairs for which *G* and *g* are within a distance of 1 Mb from each other. (**B**) The copy number-adjusted residual expression as a function of the non-adjusted expression, in log space. Shown here are the expression levels for six genes with an in-*cis* correlation ranging from 0 to 0.9. Each dot represents one breast cancer patient. The effect of copy number-adjusted-residual expression increases with increasing in-*cis* correlation level. The dotted line is the diagonal, and the solid line is the regression line.

**Figure 5:**
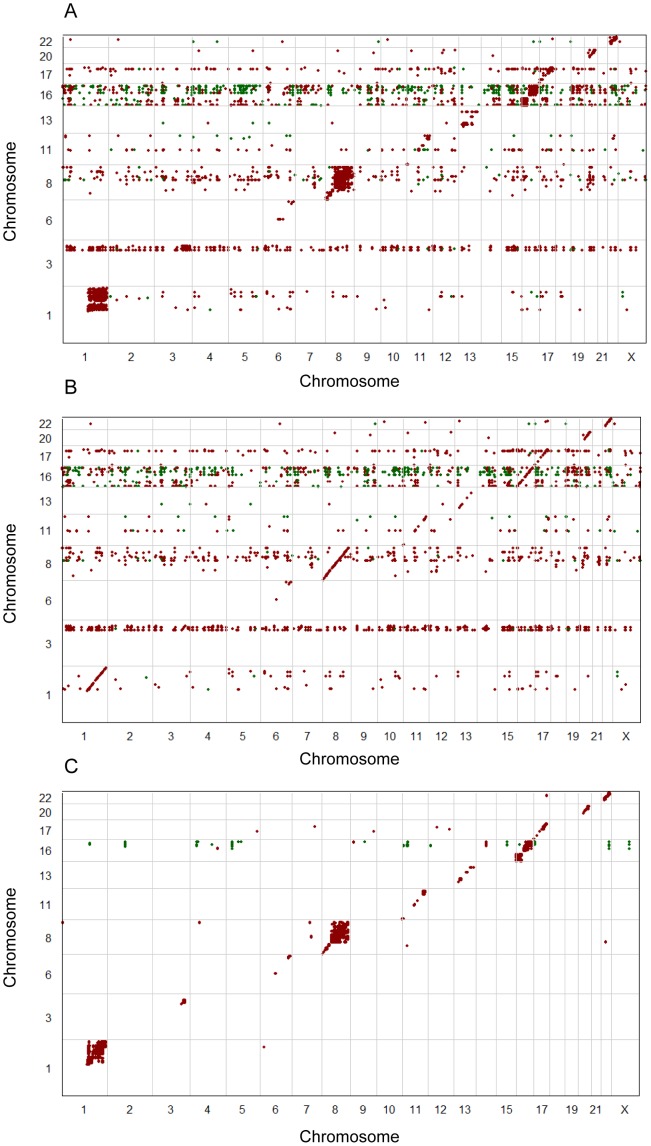
Effect of residual expression. Correlation plots showing how the level of high-level in-*trans* correlations change across the genome with and without copy number-adjusted residual expression correlation. Red dots signify positive in-*trans* Pearson correlation above 0.6, and green dots signify negative in-*trans* Pearson correlation below −0.6. The x-axis shows the genomic positions of all 25,688 genes and the y-axis represents the genomic position of the 578 in-*cis* genes. (**A**) High *in-trans* correlations between expression of in-*cis* genes to expression of all genes. (**B**) High *in-trans* correlations between expression of in-*cis* genes to residual expression of all genes. (**C**) High i*n-trans* correlations between copy number of in-*cis* genes to the expression of all genes.

The final step of the workflow led to the identification of in-*cis* genes significantly associated with at least one biological process in *trans*. For this purpose, the copy number-adjusted residual expression was calculated for all 25,688 genes. Each in-*cis* gene was taken separately as a pivot and all 25,688 genes were ranked according to the in-*trans* correlation between their copy number-adjusted residual expression and the non-adjusted expression of the pivot gene. The importance of adjusting for copy number is most pronounced for genes in close proximity (see Figure 4A), and the effect of using copy number-adjusted residual expression increases with the in-*cis* correlation (see Figure 4B and Figure 5).

**Figure 6:**
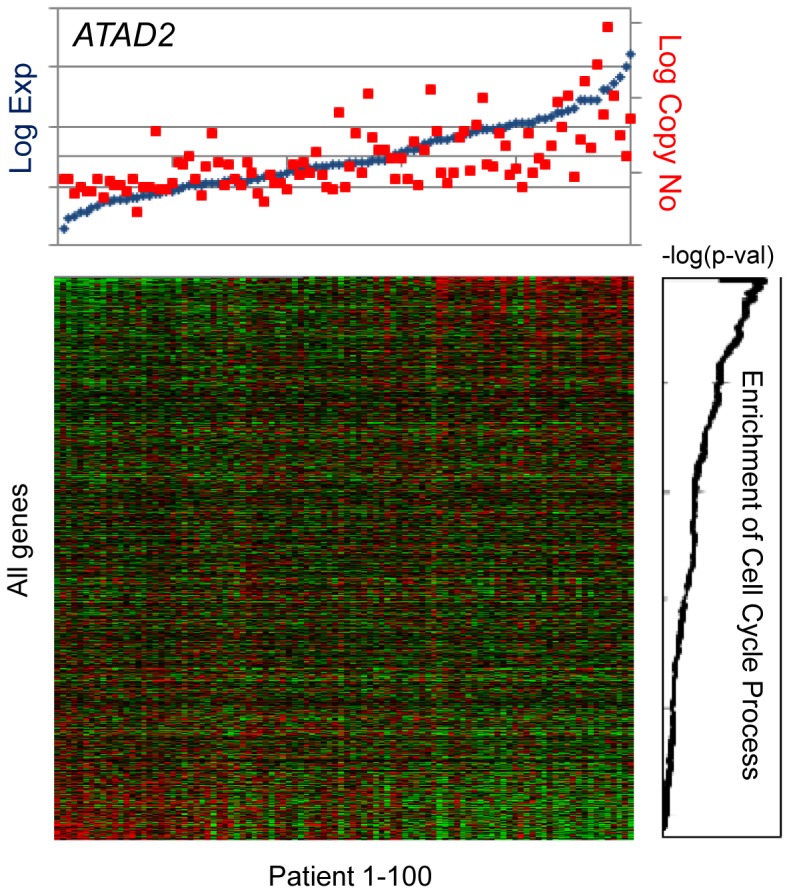
Enrichment of the Cell Cycle Process GO term in *ATAD2* correlated genes. All genes were ranked according to the level of correlation between their copy number-adjusted-residual expression profile and the expression levels of *ATAD2* (pivot for this analysis). The heatmap represents the expression levels of all 25,688 genes after ranking them according to the criteria mentioned above and after sorting the samples according to *ATAD2* expression levels. Top panel in blue and red presents the expression and copy number levels of *ATAD2* across the 100 samples, respectively. The graph shows the significance level in –log(hypergeometric p-value) of cell cycle process genes in the ranked list of genes. Optimal enrichment is attained at the top 189 genes, with 14 times more cell cycle process genes than would be expected by chance (mHG 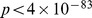).

**Figure 7:**
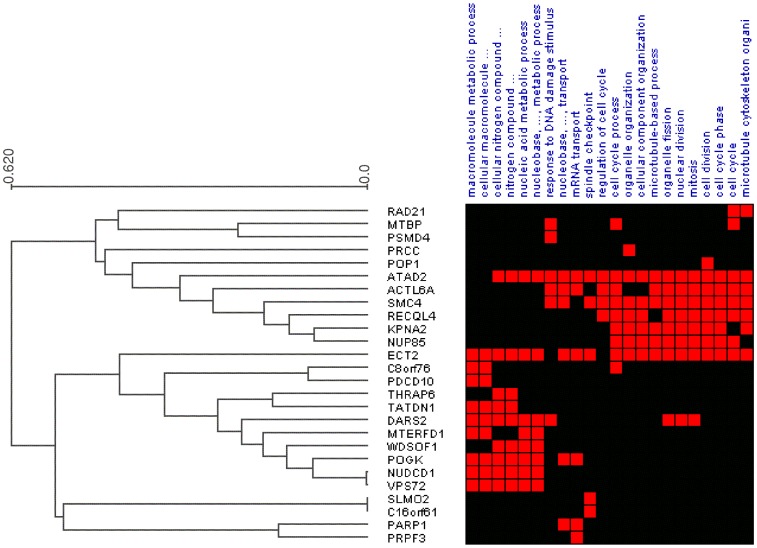
Associations between iPAC genes and traits (biological processes). A hierarchical clustered heatmap representation of traits associated with at least four iPAC genes. A red entry indicates a significant association between an iPAC gene and the corresponding trait (see Figure S4 for all the significant associations). The Expander suite [66] using average Euclidian distance was used to calculate and visualize the hierarchical clustering analysis.

Overrepresentation of Gene Ontology (GO) biological process terms in the above ranked list of genes was statistically assessed (Figure 6). Out of the 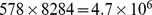 potential associations (for every in-*cis* gene and every GO term tested), we first selected those with enrichment score 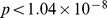 (corresponding to 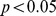 after Bonferroni correction). This resulted in 19,606 associations covering 467 GO terms and all 578 in-*cis* genes. Finally, simulations were used to call significant gene-process associations (see Materials and Methods). This yielded a total of 276 highly significant associations, covering 56 in-*cis* genes (henceforth called iPAC genes) and 97 unique GO terms (called traits) at a false discovery rate (FDR) of less than 1% (see Figure 2, Table 1 and Table S3). Cell cycle related processes commonly occurred as traits of the iPAC genes, consistent with an association to tumor development and progression related processes (see Figure 7 and Figure S4).

**Table 1:** Description and properties of the 56 iPAC genes.

Gene	Full gene name	Cytoband	Highest associated GO term (trait)	Score	Annot
*DARS2*	*aspartyl-tRNA synthetase 2, mitochondrial*	1q25.1	nucleic acid metabolic proc.	94.31	
*ATAD2*	*ATPase family, AAA domain containing 2*	8q24.13	cell cycle	91.53	BC
*SMC4*	*structural maintenance of chromosomes 4*	3q25.33	cell cycle	90.42	BC
*ACTL6A*	*actin-like 6A*	3q26.33	cell cycle	87.78	C
*RECQL4*	*RecQ protein-like 4*	8q24.3	cell cycle	86.78	BC
*ECT2*	*epithelial cell transforming sequence 2 oncogene*	3q26.31	cell cycle	82.84	C
*POGK*	*pogo transposable element with KRAB domain*	1q24.1	nucleic acid metabolic proc.	82.62	
*MTBP*	*Mdm2, transformed 3T3 cell double minute 2, p53 binding protein*	8q24.12	cell cycle	80.67	C
*VPS72*	*vacuolar protein sorting 72 homolog (S. cerevisiae)*	1q21.2	nucleic acid metabolic proc.	80.29	
*NUDCD1*	*NudC domain containing 1*	8q23.1	nucleic acid metabolic proc.	80.27	C
*MTERFD1*	*MTERF domain containing 1*	8q22.1	nucleic acid metabolic proc.	78.77	
*WDSOF1*	*DDB1 and CUL4 associated factor 13*	8q22.3	nucleic acid metabolic proc.	78.14	BC
*NUP85*	*nucleoporin 85kDa*	17q25.1	cell cycle	75.28	
*RAD21*	*RAD21 homolog (S. pombe)*	8q24.11	cell cycle	74.77	BC
*KPNA2*	*karyopherin alpha 2 (RAG cohort 1, importin alpha 1)*	17q24.2	cell cycle proc.	74.09	BC
*C8orf76*	*chromosome 8 open reading frame 76*	8q24.13	cell cycle proc.	69.94	
*POP1*	*processing of precursor 1, ribonuclease P/MRP subunit*	8q22.2	cell division	64.40	C
*TATDN1*	*TatD DNase domain containing 1*	8q24.13	cellular macromol. metabolic proc.	61.06	BC
*PDCD10*	*programmed cell death 10*	3q26.1	cellular macromol. metabolic proc.	58.81	C
*THRAP6*	*mediator complex subunit 30*	8q24.11	cellular nitrogen compound metab. proc.	55.46	BC
*RPL30*	*ribosomal protein L30*	8q22.2	cellular macromolecule biosynth. proc.	46.14	C
*PRCC*	*papillary renal cell carcinoma (translocation-associated)*	1q23.1	organelle organization	38.66	C
*C1orf35*	*chromosome 1 open reading frame 35*	1q42.13	chromosome organization	34.69	C
*PARP1*	*poly (ADP-ribose) polymerase 1*	1q42.12	chromosome organization	34.10	BC
*MRPS23*	*mitochondrial ribosomal protein S23*	17q23.2	positive regulation of ligase activity	28.47	C
*PSMD4*	*proteasome (prosome, macropain) 26S subunit, non-ATPase, 4*	1q21.2	response to DNA damage stimulus	27.44	
*SETDB1*	*SET domain, bifurcated 1*	1q21.2	chromatin modification	23.51	C
*HNRPU*	*heterogeneous nuclear ribonucleoprotein U*	1q44	chromatin modification	20.52	C
*BOP1*	*block of proliferation 1*	8q24.3	DNA conformation change	17.27	C
*SIAHBP1*	*poly-U binding splicing factor 60KDa*	8q24.3	mitotic sister chromatid segregation	16.14	C
*PRPF3*	*PRP3 pre-mRNA processing factor 3 homolog (S. cerevisiae)*	1q21.2	mRNA transport	15.77	C
*PPM1D*	*protein phosphatase, Mg2+/Mn2+ dependent, 1D*	17q23.2	mitotic cell cycle checkpoint	14.29	BC
*FAM33A*	*spindle and kinetochore associated complex subunit 2*	17q23.2	mitotic cell cycle checkpoint	14.12	BC
*MRPL9*	*mitochondrial ribosomal protein L9*	1q21.3	establishment of organelle localization	13.74	
*C22orf28*	*chromosome 22 open reading frame 28*	22q12.3	cellular protein metabolic proc.	13.41	
*SLMO2*	*slowmo homolog 2 (Drosophila)*	20q13.32	spindle checkpoint	11.92	
*CHRAC1*	*chromatin accessibility complex 1*	8q24.3	mitotic metaphase plate congression	11.91	C
*C16orf61*	*chromosome 16 open reading frame 61*	16q23.2	spindle checkpoint	11.28	BC
*ISG20L2*	*interferon stimulated exonuclease gene 20kDa-like 2*	1q23.1	DNA-dependent DNA replication init.	11.07	
*CSNK1E*	*casein kinase 1, epsilon*	22q13.1	neural tube development	10.25	BC
*FAM91A1*	*family with sequence similarity 91, member A1*	8q24.13	establishment of mitotic spindle loc.	10.08	
*TOMM20*	*translocase of outer mitochondrial membrane 20 homolog (yeast)*	1q42.3	transcription	10.03	
*C20orf20*	*chromosome 20 open reading frame 20*	20q13.33	mitotic cell cycle spindle checkpoint	9.67	C
*GALNS*	*galactosamine (N-acetyl)-6-sulfate sulfatase*	16q24.3	carbohydrate catabolic proc.	9.05	BC
*AZIN1*	*antizyme inhibitor 1*	8q22.3	histone mRNA metabolic proc.	8.19	C
*MTL5*	*metallothionein-like 5, testis-specific (tesmin)*	11q13.2	water-soluble vitamin biosynthetic proc.	−8.37	
*TPD52*	*tumor protein D52*	8q21.13	regeneration	−8.57	BC
*ARID4B*	*AT rich interactive domain 4B (RBP1-like)*	1q42.3	organ regeneration	−8.80	C
*THC2340878*	*NA*	8q13.2	programmed cell death	−9.06	
*CHTOP*	*chromatin target of PRMT1*	1q21.3	activation of plasma proteins	−11.73	
*TMEM70*	*transmembrane protein 70*	8q21.11	regulation of Rho protein signal transd.	−13.02	BC
*DPM1*	*dolichyl-phosphatemannosyltransferase polypeptide 1, cat. subunit*	20q13.13	negative regulation of gene expression	−13.36	C
*PYCRL*	*pyrroline-5-carboxylate reductase-like*	8q24.3	membrane invagination	−15.15	
*IMPAD1*	*inositol monophosphatase domain containing 1*	8q12.1	positive regulation of cell death	−15.88	
*STX16*	*syntaxin 16*	20q13.32	cellular protein metabolic process	−15.88	
*PIGM*	*phosphatidylinositol glycan anchor biosynthesis, class M*	1q23.2	response to external stimulus	−22.09	

Scores in the table are the negative logarithms of the enrichment scores, the sign indicating whether the association of the trait to the genes is positively or negatively correlated with the iPAC gene. The annotation column indicates genes previously linked with breast cancer (BC) and among those that are not, genes linked to cancer in general (C), based on annotation of the genes obtained with IPA (Ingenuity® Systems, www.ingenuity.com).

### Properties of the identified iPAC genes

Four of the 56 iPAC genes were commonly deleted and 52 commonly amplified. The iPAC genes encode proteins with various biological roles, including enzymes, regulators of transcription and translation, and transporter molecules (Figure S3B). Five of them were themselves members of the biological process(es) they were found to be associated with (*MTBP, RAD21*, *RECQL4*, *SETDB1,* and *SMC4)*. Comparing the 56 iPAC genes against the background of all other genes using GOrilla, the 56 genes were found to be associated to four biological processes all commonly disrupted in cancer: cell cycle, cell cycle process, nucleic acid metabolic process, and chromosome organization (Table S4 and Figure S5).

Among the iPAC genes, 38 mapped to chromosomes 1 (n = 16) and 8 (n = 22), while the rest were located on chromosomes 3, 11, 16, 17, 20, and 22 (Figure 2 and Figure S6). There was a tendency for iPAC genes to reside in blocks of commonly aberrant segments. As would be expected by their mutual proximity and their high in-*cis* correlation, the expression levels of iPAC genes residing in the same block were highly correlated (Figure S7). Accordingly, the scope for further narrowing down the list of candidates based on copy number and expression data alone was limited. However, in several cases, iPAC genes in close proximity were found to be associated with different biological processes. For example, *PRPF3* and *SETDB1* are less than 1 Mb apart from each other and were associated with mRNA transport and chromatin modification, respectively.

Figure S8 shows how the patient samples clustered according to the expression of the iPAC genes. Most of the luminal samples clustered together, as did the basal-like samples, with the latter having a tendency towards higher expression of the iPAC genes. We note that the expression levels of the iPAC genes were not found to be significantly associated with survival (data not shown).

### Knockdown experiment with siRNA

To investigate the effect of the selected iPAC genes on cell viability, siRNA knockdown was performed for three iPAC genes (*ECT2*, *PSMD4* and *MTBP*) in two breast cancer cell lines (MCF7 and MDA-MB-231; see the Supporting Information uploaded to the file inventory: File S1.pdf). For one of the siRNAs tested against *ECT2* we observed a ∼30% reduction in cell viability (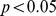, Figure S9) in the MCF7 cell line. ECT2 is a guanine nucleotide exchange factor for Rho family GTPases and was most strongly associated with the cell cycle GO term and amplified in 15% of the samples. The reduced cell viability after knockdown emphasizes the importance of the iPAC gene *ECT2* in the MCF7 cell line. A smaller reduction in cell viability was also observed for *PSMD4* (data not shown).

### Robust iPAC signature in a validation cohort

In order to validate the robustness of the 56 identified genes, we investigated their iPAC characteristics in an independent breast cancer cohort (UNC), consisting of 73 patients [25]. Out of the 56 iPAC genes identified in our study, 51 were among the genes measured in the UNC study. The five remaining genes (*IMPAD1*, *FAM33A*, *FAM91A1*, *PARP1* and *THC2340878*) had been removed further upstream in the analysis and data preprocessing in the UNC study.

**Figure 8:**
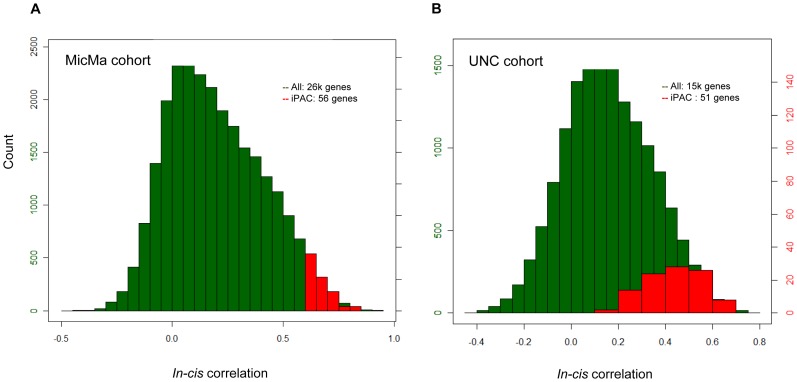
Distribution of in-*cis* correlation levels between copy number and expression in the MicMa and UNC cohorts. Green bins in the histogram show distribution of in-*cis* correlation levels of all genes in the data set, while red bins show the distribution for only the identified iPAC genes. The left-hand y-axes in each histogram show the count in each bin among all genes, and the right-hand axes show the count for iPAC genes in each bin. (**A**) Distribution of the in-*cis* correlation levels in the MicMa cohort. (**B**) Distribution of the in-*cis* correlation levels in the UNC cohort. The iPAC genes were inferred from the MicMa cohort.

**Figure 9:**
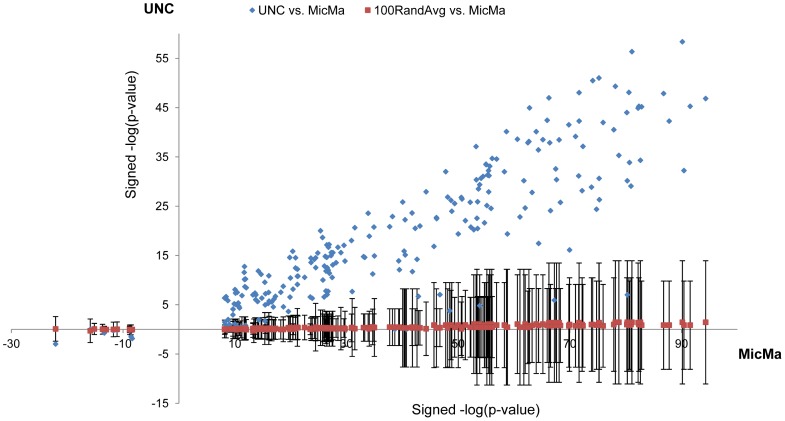
Association consistency of iPAC genes in the validation cohort. Blue dots represent associations between an iPAC gene and a GO term. The blue dots are plotted according to the level of association, as signed –log(p-value), in the MicMa cohort (x-axis) and in the UNC cohort (y-axis), where signed –log(p-value) refers to –log(mHG p-value) for positive associations and log(mHG p-value) for negative associations. A monotone relation is observed, supporting the iPAC behavior of the MicMa inferred iPAC genes in the validation cohort. A bar with a red dot in the center is plotted for each blue dot representing 1 standard deviation (SD) of the associations generated by associating 100 random genes from the UNC cohort to the relevant GO term.

The in-*cis* correlation between copy number and expression for the iPAC genes ranged from 0.16 (*SETDB1*) to 0.69 (*PPM1D*) in the validation cohort, with an average of 0.43 (Figure 8). To assess the in-*trans* associations for the iPAC genes in the validation cohort, all genes were ranked according to the level of correlation between their copy number-adjusted residual expression and the expression of each iPAC gene in the validation cohort. The association between each iPAC gene and each of the 97 GO terms identified in the original analysis was then assessed. The results showed high level of consistency of enrichments between the two cohorts (Figure 9). As a further confirmation of consistency, we compared these results to the association levels (negative logarithms of the enrichment scores) of 100 random genes to each of the above GO terms. It was found that 95% of the iPAC gene/trait pairs tested in the validation cohort had association levels exceeding 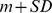, where 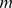 is the average and 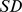 is the standard deviation of association levels obtained for the random genes, and 80% of the pairs had association levels exceeding 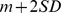 (Figure 9). This shows that the level of association of the iPAC genes with their relevant biological process (represented by the GO term) in the validation cohort is not random.

## Discussion

Copy number aberrations are common in breast cancer, but to what extent such aberrations affect cancer cell phenotype through alterations of the transcriptional program is not yet known. The methods we propose here aim to identify genes subject to selection in breast cancer by detecting commonly aberrant genes affected on the gene expression level by genomic aberrations. Furthermore, the method requires the identified genes to be correlated with genes collectively enriched with respect to GO biological processes. Thus, it is through the influence on other genes and their associated processes that the iPAC genes are identified.

### Integrative analysis as a tool for inferring causality

Numerous high-throughput profiling expression studies have identified clusters of genes with expression varying in a coordinated manner over time or across disease states. However, such studies generally give no information about the directionality of gene interactions unless additional information is available. Strong association between the mRNA expression levels of two genes may result from one gene regulating the other, both being regulated by a common factor, or a combination of both. The combined use of copy number and expression data allows the distinction between a situation where the expression of one gene influences the expression of another gene and a situation where the expression levels of the two genes are merely correlated [17].

### Relationship to other methods

Several strategies that aim to identify driver genes in cancer exploit the integration of matched copy number and expression data. Woo *et al*. [23] worked with an integrated copy number and expression data set and used the prognostic significance of genes to guide the selection process. Akavia *et al*. [17] also utilized this sort of integration in their CONEXIC algorithm. Their study assumed that a driver mutation would occur more often than by chance in multiple tumors, that the mutation would be correlated with the expression of a group of genes (a module), and that copy number changes often had an effect on expression of the driver that thus further influenced the expression of the module [17]. The CONEXIC approach is founded on the notion that the expression levels of the driver, rather than the determinants of that expression level, confers a fitness advantage to the tumor. Alteration of copy number is only one way of achieving this, manifested by a high frequency of aberrations in a patient cohort.

The iPAC approach has a similar rationale as CONEXIC. However, our method differs from the approaches described above in several aspects. First, we use residual expression for the in-*trans* correlation analysis, thus bypassing the potential confounder effect of co-occurring copy numbers. Second, we use a robust enrichment analysis approach to identify aberrations that lead to a significant shift in cancer-related transcriptional programs. Using the enrichment framework, we assign statistical significance to gene-process associations. By taking advantage of the residual expression, the modulator properties of the iPAC genes are more robustly captured. Such modulator effects on biological processes interrupted in cancer may go beyond the direct effects on a pathway; transcriptional responses launched by the cell after physiologic alterations may result from various indirect influences and mechanisms [17], and in this respect, the iPAC genes represent a diverse set of candidates.

### Characteristics of the iPAC genes

The list of iPAC genes includes 16 genes previously associated with breast cancer and 20 additional genes associated with cancer in general (Table 1). For example, *ATAD2* was highly associated to the cell cycle process, indicating that the cell cycle module is activated when *ATAD2* is amplified and overexpressed. *ATAD2* is an ATPase and was recently reported to be a cofactor for the *MYC* oncogene [40]. While copy number was a predominant determinant of *ATAD2* expression levels, other factors also probably influence *ATAD2* expression levels and through its expression level, *ATAD2* is proposed to affect its target process. Another example is *TPD52* which was highly associated to regeneration; this gene has previously been suggested as a potential driver gene and reported amplified and overexpressed in various cancer types, including breast cancer [41], [42], [43], [44]. Furthermore, *PPM1D* was strongly associated to the mitotic cell cycle checkpoint; this gene encodes a serine/threonine phosphatase, maps to the 17q23.2 amplicon and has been shown to be involved in the regulation of several tumor suppressor pathways, including the p53 pathway [45], [46]. Amplification of this gene has previously been found to be correlated with overexpression in breast cancer [47]. The iPAC gene *KPNA2* was associated with the trait of nuclear division, and is a member of the importin family of proteins involved in nuclear transport. *KPNA2* has been proposed to be a prognostic marker in breast cancer [48], and overexpression of this gene has been associated with poor prognosis, expression signatures of high proliferation, and tumor grade [49], [50].

The iPAC genes also include several genes not previously associated with cancer. One interesting example is the gene *MTL5* which was negatively correlated with the water-soluble vitamin biosynthetic process and encodes a protein with homology to the metal-binding motif of the metallothionein (MT) family [51]. *MTL5* is located on chromosome 11q13.2 and was found amplified in 17% of our investigated breast cancer samples. Through their ability to bind metal, MT proteins can affect the activity of several proteins and enzymes dependent on metals as co-factors. In this respect, MT proteins play important roles in apoptosis and proliferation [52]. Furthermore, elevated expression of MT proteins has been reported in various cancer types, including breast cancer [52], [53], [54] and was also linked to modulation of p53 activity through zinc exchange [55], [56]. Dividing our samples according to p53 mutational status, *MTL5* was one of the top 2% most down regulated genes in mutated p53 (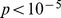) (data not shown). As *MTL5* was found to be amplified in a significant proportion of the samples in our cohort, and because of its iPAC properties, our results indicate that the gene may have an important role in breast cancer, similar to the homologus MT proteins. Many homologs of *MTL5* exist both in animals and plants, suggesting that the function of this gene is conserved [57].

### Proof-of-concept knockdown experiments

We selected three iPAC genes for siRNA knockdown experiments. Out of these, silencing of *ECT2* led to significant decrease in cell viability. By using our approach, this gene was found to be most highly associated with cell cycle related traits. The protein ECT2 has been shown to regulate cytokinesis [58], which can explain the effect on cell viability after knockdown. *ECT2* has been found to be up-regulated during transition to malignancy in a mouse model [59], to be amplified and overexpressed in non-small cell lung cancer [60], and to have an elevated expression in colorectal cancer [61].

In another study, siRNA-mediated knockdown of the iPAC gene *RAD21* was found to decrease cell growth and enhance cytotoxicity in MCF7 and T47D breast cancer cell lines [62]. *RAD21* encodes a phosphoprotein and is a component of the cohesin complex essential for chromosome segregation during mitosis/meiosis and DNA repair [63], [64]. In our breast cancer cohort, *RAD21* was found to be amplified in 36% of the tumor samples and to be highly associated with the cell cycle trait. Strong association to cell cycle has been shown to correlate with cell proliferation for the same patient samples [26], and enhanced expression of this protein has been associated with poor prognosis and resistance to chemotherapy in breast cancers [65].

### Conclusion

Whole-genome integrative analyses of copy number and gene expression data is a useful tool in genome-wide searches for candidate driver genes in cancer. The first phase of analysis is typically to detect genes with frequent aberrations in copy number and strong in-*cis* correlation to gene expression. For example, in our study, the gene *ERBB2* was ranked 7 out of 6373 genes with respect to the in-*cis* correlation level, indicating a direct link between copy number and expression. However, even among those genes that satisfy these criteria there are potentially many passengers with no direct oncogenic role. In the opposite direction, there may be genes that manifest moderate in-*cis* expression but still drive cancer-related processes through their expression level. Regulation of these expression levels may be selected for in the cancer through copy number changes as well as other mechanisms (e.g. altered methylation). Our aim has been to detect genes for which the gene-gene correlation structure of the expression data reveals additional evidence to support a link to a phenotype. The iPAC gene *ATAD2*, which was ranked only 450 in the in-*cis* correlated genes and hence would easily have been missed by in-*cis* focused methods, illustrates this point. Several similar examples are described above, indicating that the iPAC procedure does indeed capture biologically relevant genes not found on the top of the list of in-*cis* correlated genes.

Validation in an independent cohort of the proposed methodology and of the observation regarding the 56 iPAC genes found in our initial analysis supports method robustness and justify focus on the identified genes with respect to their tumorigenic role. In this study, we have selected GO biological process terms as they represent a comprehensive view of functional traits. It is clearly possible to select other annotation approaches for this purpose. For example, one could assess the enrichment of molecular pathways or transcription factor networks among the in-*trans* correlated genes. We provide cell line based experimental data for the effect of *ECT2* on cell viability; however, further functional validation is still needed to firmly establish the role of the 56 iPAC genes in breast cancer.

The framework for the identification of in-*trans* regulatory mechanisms, as exemplified here in human breast cancer, is applicable to any kind of data with existing comparable aCGH, expression profiles and a collection of gene sets representing transcriptional programs. We propose this method as an unbiased and robust approach for the identification of genes of relevance to tumorigenesis.

## Supporting Information

Figure S1 **Copy number and expression correlations.** (**A**) Pearson correlation of copy number data for all the 25,688×25,688 genes. (**B**) Pearson correlation of copy number and expression of all 25,688×25,688 genes, with in-*cis* correlation along the diagonal. Color map represents the Pearson correlation coefficient.
(TIF)

Figure S2 **GO terms enriched among the in-** ***cis*** **correlated genes.** The GO biological process statistical enrichment analysis was performed by GOrilla. The input for GOrilla in this analysis was the list of 6373 commonly aberrant genes ranked according to their in-*cis* correlation.
(TIF)

Figure S3 **Functional annotation of genes.** (**A**) The 578 in-*cis* genes; (**B**) The 56 iPAC genes. The genes were annotated using IPA (Ingenuity® Systems, www.ingenuity.com).
(TIF)

Figure S4 **Associations between iPAC genes and their traits (GO terms).** Extension of Figure 7. A hierarchical clustered heatmap representation of all significant associations between iPAC genes and biological processes. A red entry indicates a significant association between an iPAC gene and the corresponding traits. The Expander suite [66] using average Euclidian distance was used to calculate and visualize the hierarchical clustering analysis.
(TIF)

Figure S5 **Statistical enrichment analysis of the 56 iPAC genes for GO biological processes.** Performed by GOrilla, on the list of 56 iPAC genes, compared to a background gene list consisting of all the remaining genes.
(TIF)

Figure S6
**Sample-wise genomic copy number aberrations.** Copy number aberrations are shown for chromosomes harboring at least one iPAC gene. The x-axis represents chromosomal location and the y-axis represents sample no (1–100). Green lines are regions of loss (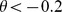), and red lines are regions of gain (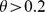). The vertical black lines indicate the locations of the 56 iPAC genes.(TIF)

Figure S7 **Correlation plots.** (**A**) Pairwise correlations of log copy number of the 56 iPAC genes. (**B**) Pairwise correlations of log expression levels of the 56 iPAC genes. Chromosomes are indicated with numbers.
(TIF)

Figure S8 **Hierarchical clustering of the expression levels of the 56 iPAC genes.** Samples are color-coded according to gene expression subtype. The clustering was made with Pearson correlation using Ward linkage. Three samples could not be subtyped and were omitted from the analysis. Color map represents log expression values.
(TIF)

Figure S9 **siRNA knockdown of the iPAC gene** ***ECT2*** **.** (**A**) Effect of siRNA knockdown of *ECT2* on cell viability in the MCF7 cell line. Four various siRNAs against *ECT2* were tested in addition to controls (bars show SD from eight replicates). The ECT2_5 siRNA shows a statistically significant reduction in cell viability compared to the non-transfected cells (asterisk; Student's t-test, p<0.05). (**B**) Relative quantification (RQ) of *ECT2* mRNA after siRNA transfections (9 replicates), showing the specificity of the knockdown in the MCF7 cell line. The data were normalized to the control (cells + transfection lipid).
(TIF)

Table S1 **GOrilla results from ranking the 6373 commonly aberrant genes.**
(XLSX)

Table S2 **The 578 in** ***-cis*** **genes.**
(XLSX)

Table S3 **Description of the 56 iPAC genes.**
(XLSX)

Table S4 **GOrilla results from 56 iPAC genes versus background gene set.**
(XLSX)

File S1 **Cell culture and siRNA transfection.**
(PDF)
