# Supplementary figures and images for: Identifying In-Trans Process Associated Genes in Breast Cancer by Integrated Analysis of Copy Number and Expression Data
Source: PLoS One. 2013 Jan 30;8(1):e53014. doi: 10.1371/journal.pone.0053014 (PMC3559658; doi:10.1371/journal.pone.0053014)

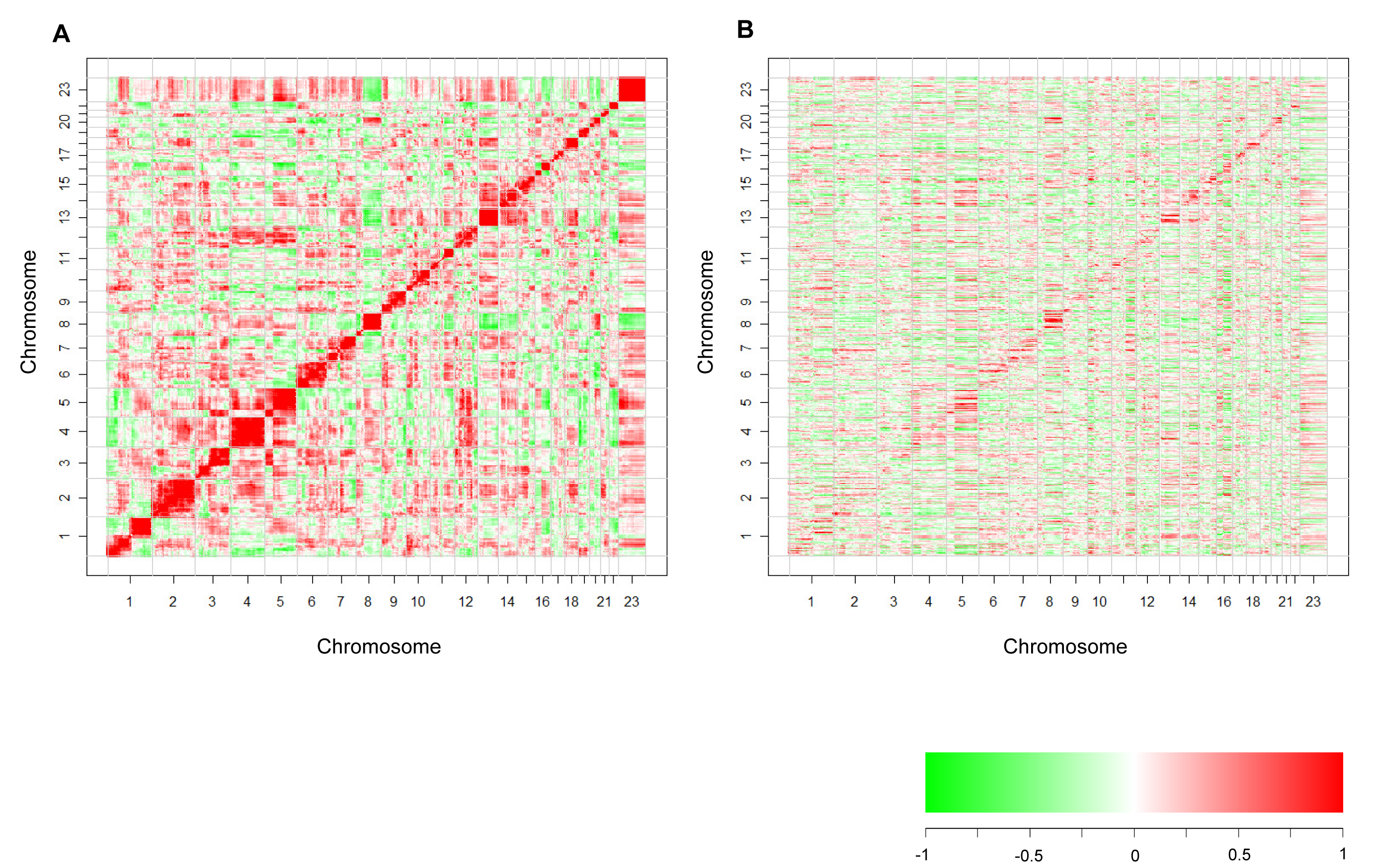

Supplement: Figure S1 — Copy number and expression correlations. (A) Pearson correlation of copy number data for all the 25,688×25,688 genes. (B) Pearson correlation of copy number and expression of all 25,688×25,688 genes, with in-cis correlation along the diagonal. Color map represents the Pearson correlation coefficient. (TIF) [file pone.0053014.s001.tif]

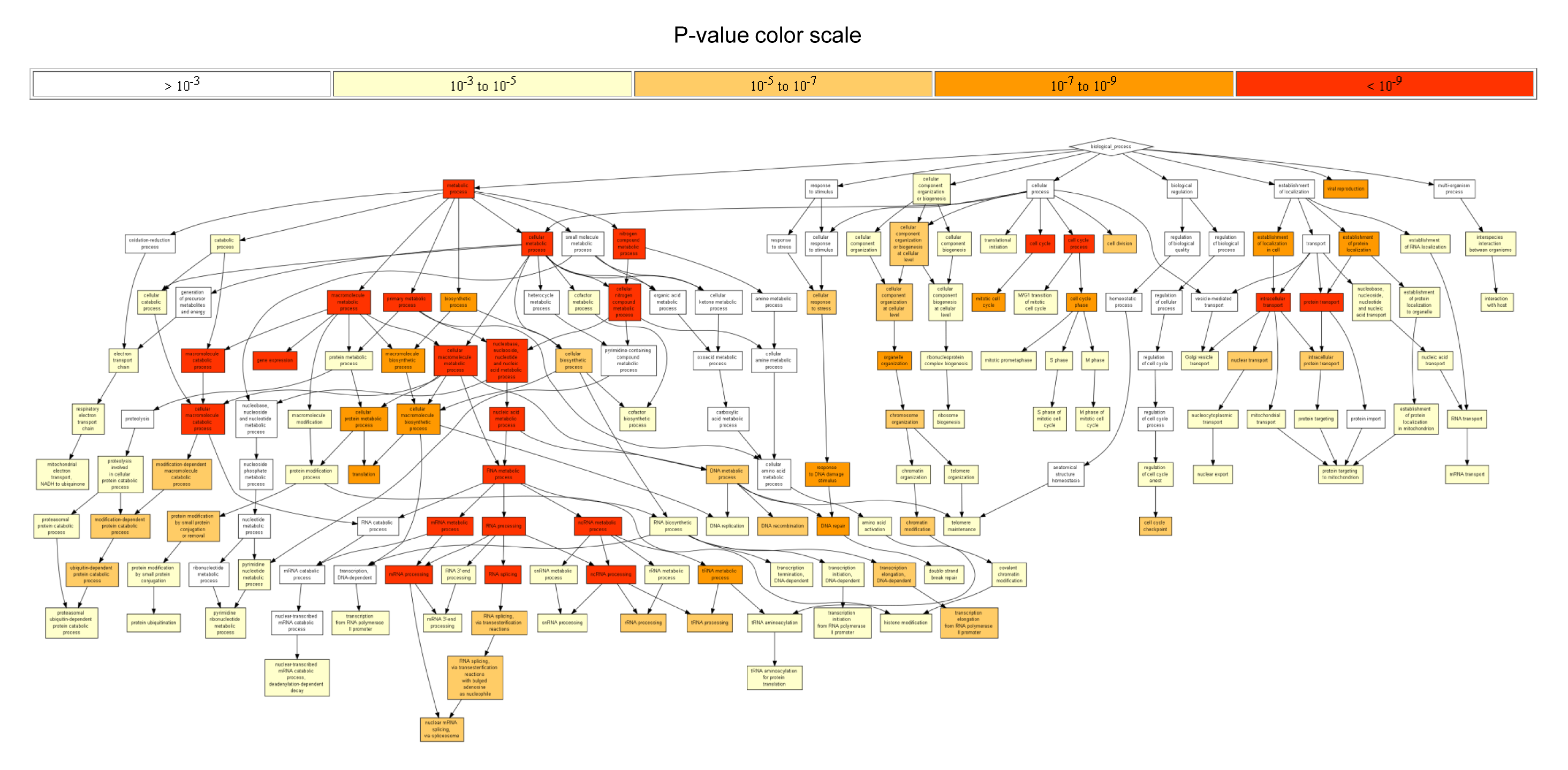

Supplement: Figure S2 — GO terms enriched among the in- cis correlated genes. The GO biological process statistical enrichment analysis was performed by GOrilla. The input for GOrilla in this analysis was the list of 6373 commonly aberrant genes ranked according to their in-cis correlation. (TIF) [file pone.0053014.s002.tif]

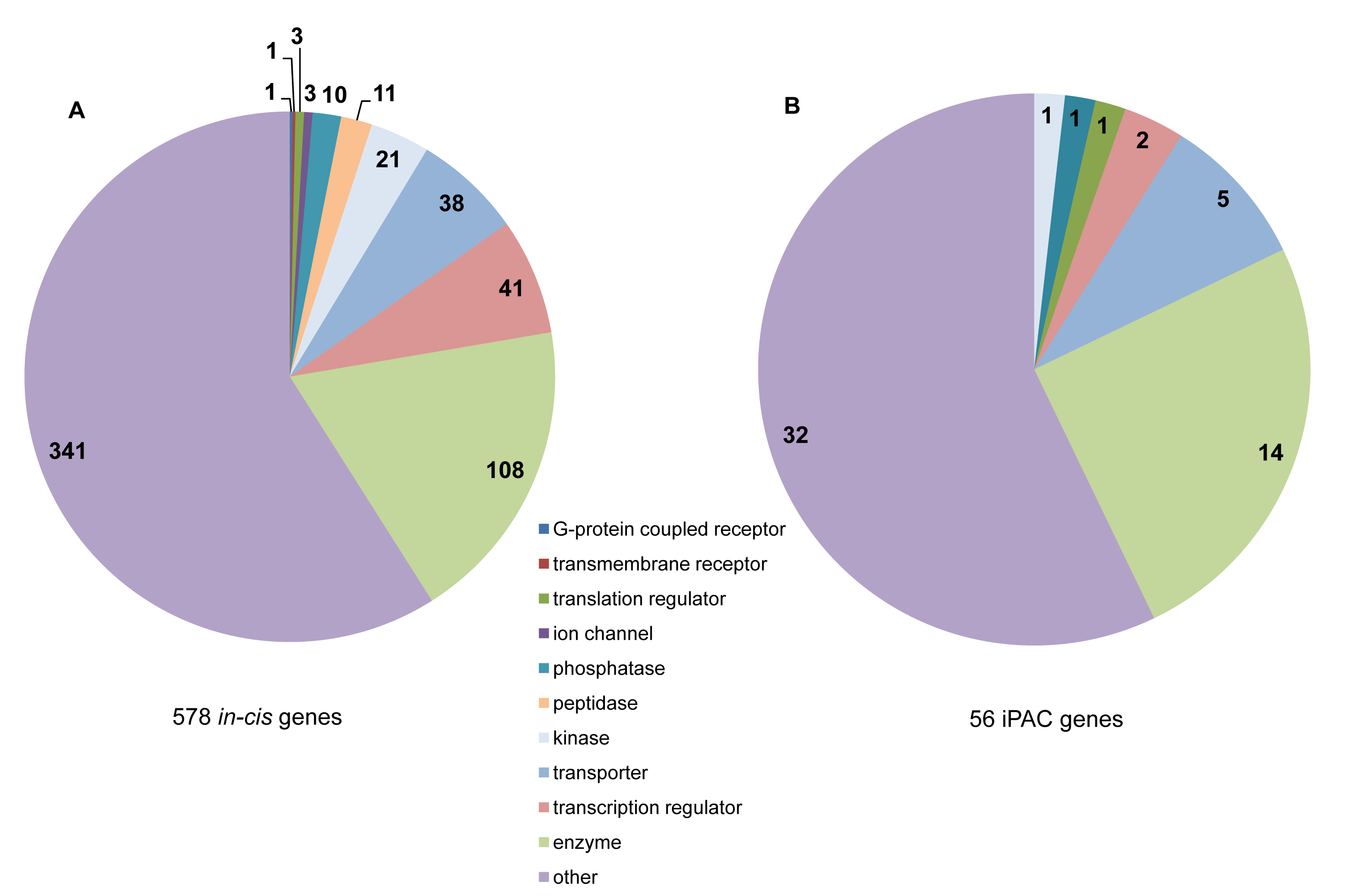

Supplement: Figure S3 — Functional annotation of genes. (A) The 578 in-cis genes; (B) The 56 iPAC genes. The genes were annotated using IPA (Ingenuity® Systems, www.ingenuity.com). (TIF) [file pone.0053014.s003.tif]

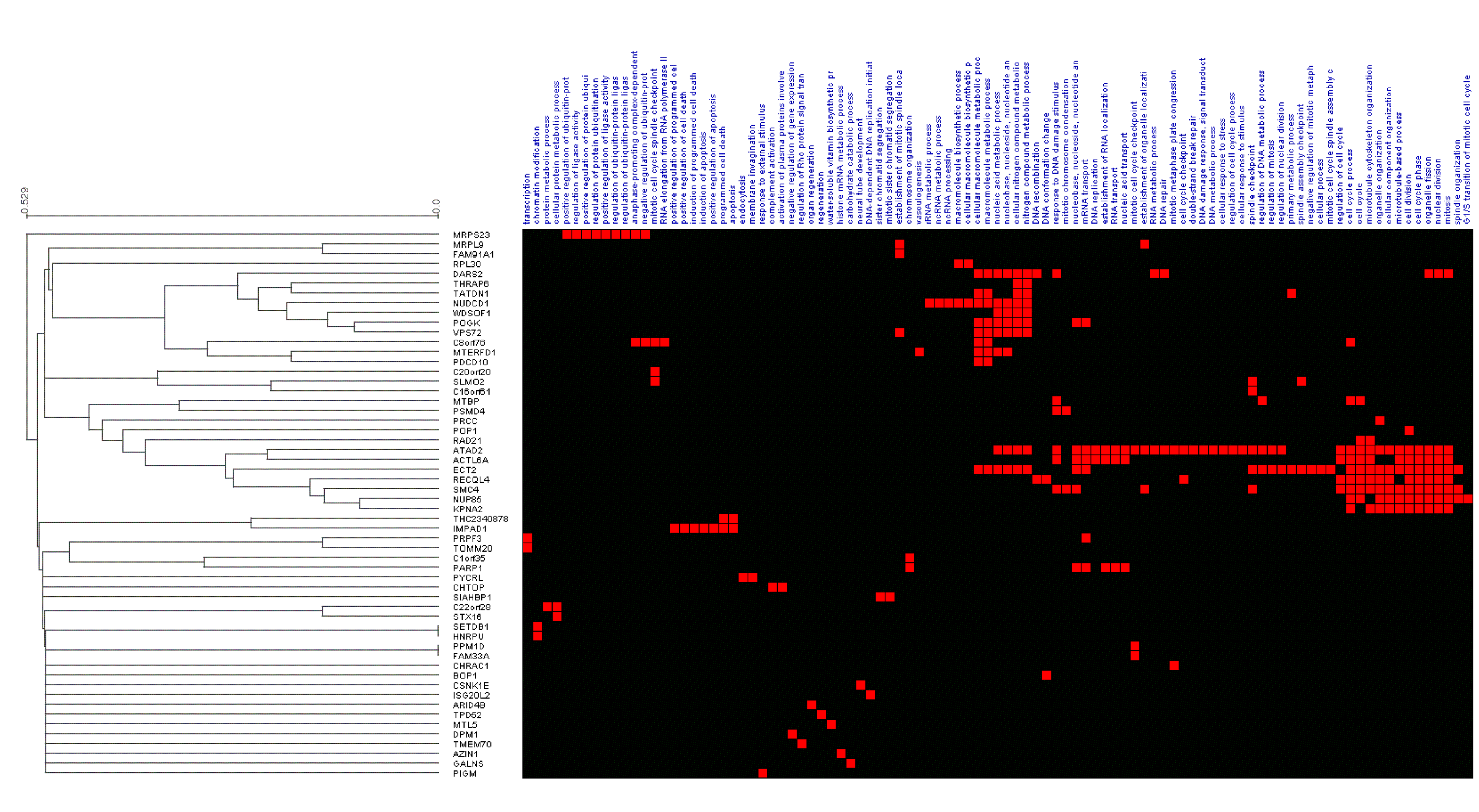

Supplement: Figure S4 — Associations between iPAC genes and their traits (GO terms). Extension of Figure 7. A hierarchical clustered heatmap representation of all significant associations between iPAC genes and biological processes. A red entry indicates a significant association between an iPAC gene and the corresponding traits. The Expander suite [66] using average Euclidian distance was used to calculate and visualize the hierarchical clustering analysis. (TIF) [file pone.0053014.s004.tif]

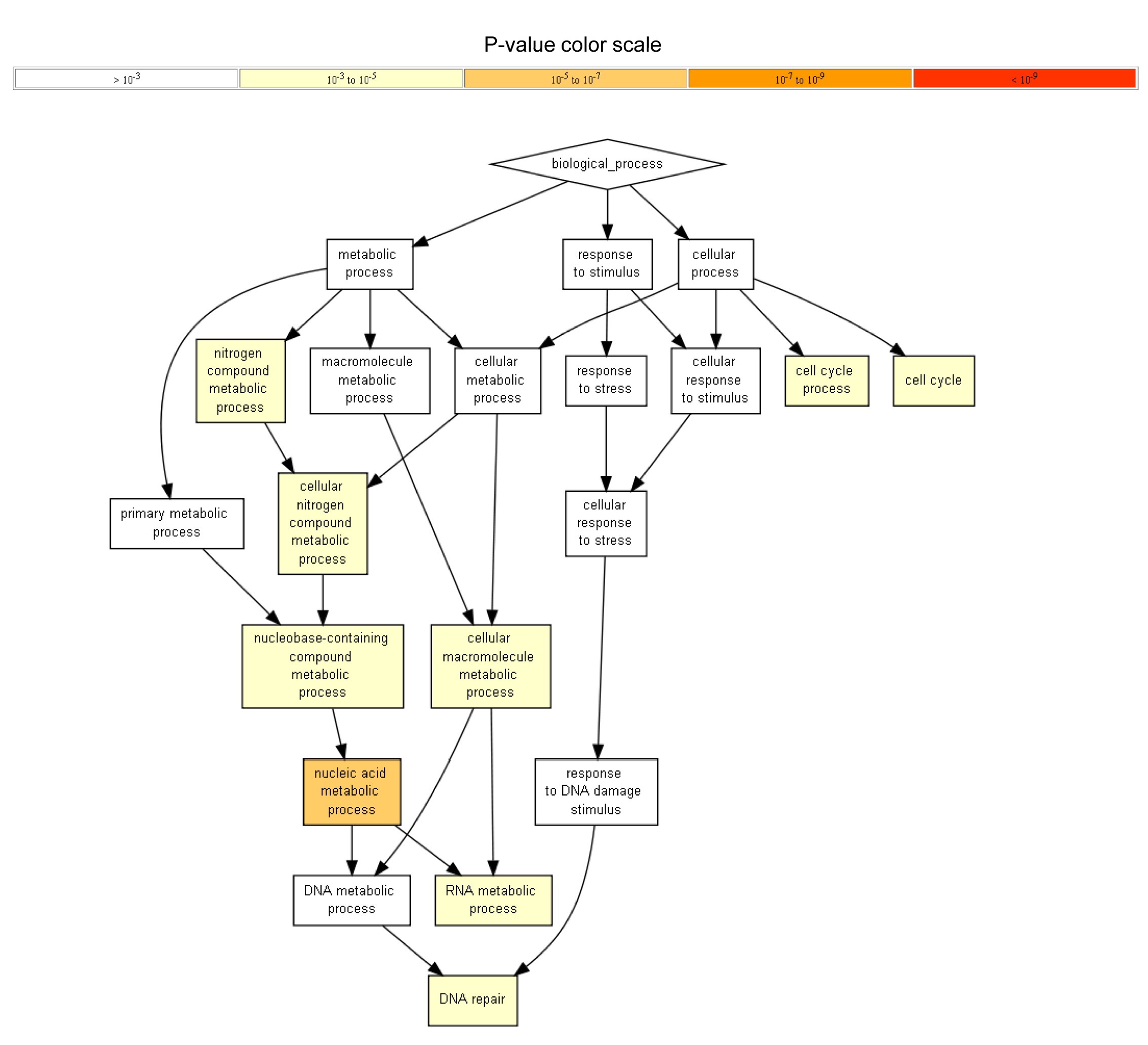

Supplement: Figure S5 — Statistical enrichment analysis of the 56 iPAC genes for GO biological processes. Performed by GOrilla, on the list of 56 iPAC genes, compared to a background gene list consisting of all the remaining genes. (TIF) [file pone.0053014.s005.tif]

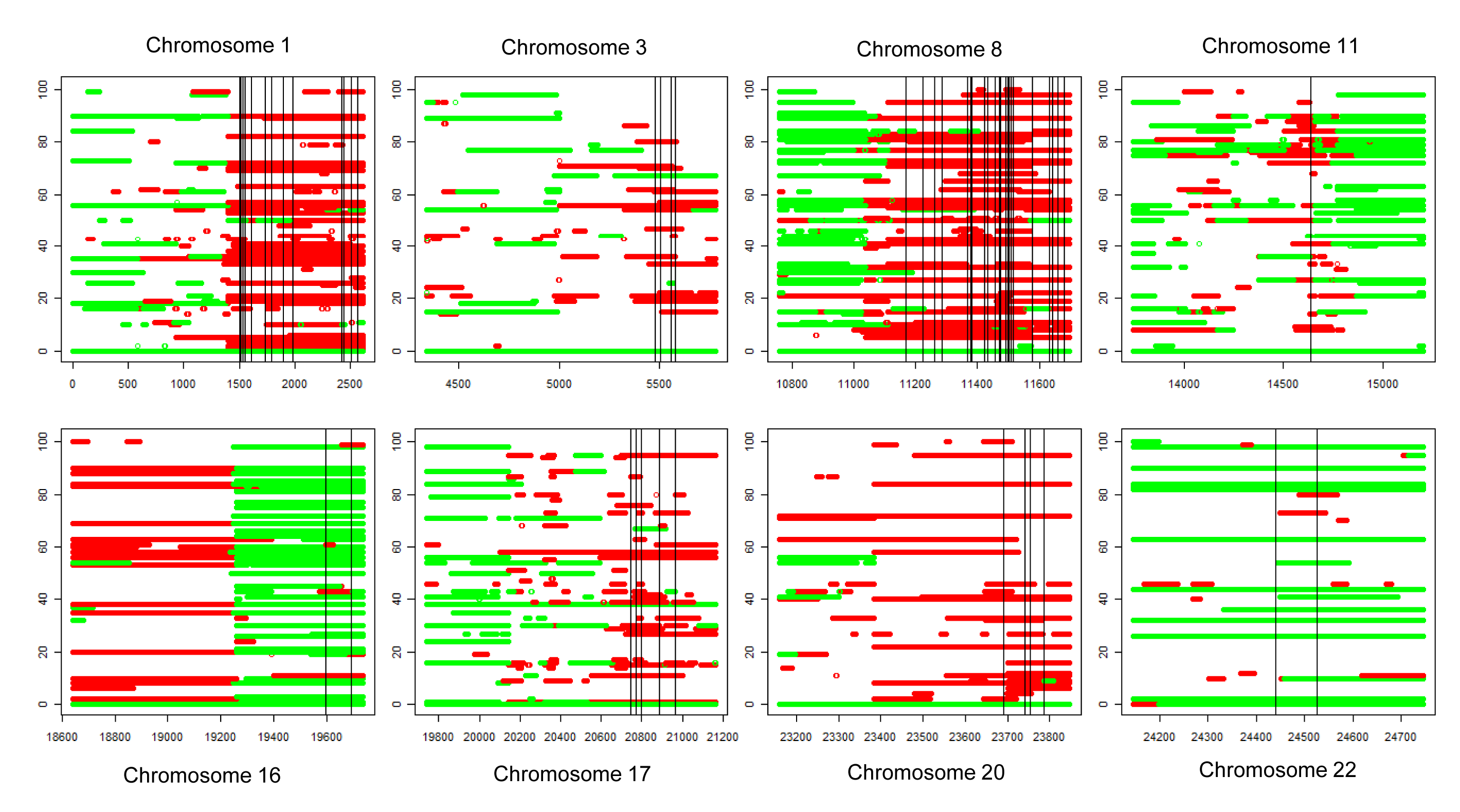

Supplement: Figure S6 — Sample-wise genomic copy number aberrations. Copy number aberrations are shown for chromosomes harboring at least one iPAC gene. The x-axis represents chromosomal location and the y-axis represents sample no (1–100). Green lines are regions of loss (), and red lines are regions of gain (). The vertical black lines indicate the locations of the 56 iPAC genes. (TIF) [file pone.0053014.s006.tif]

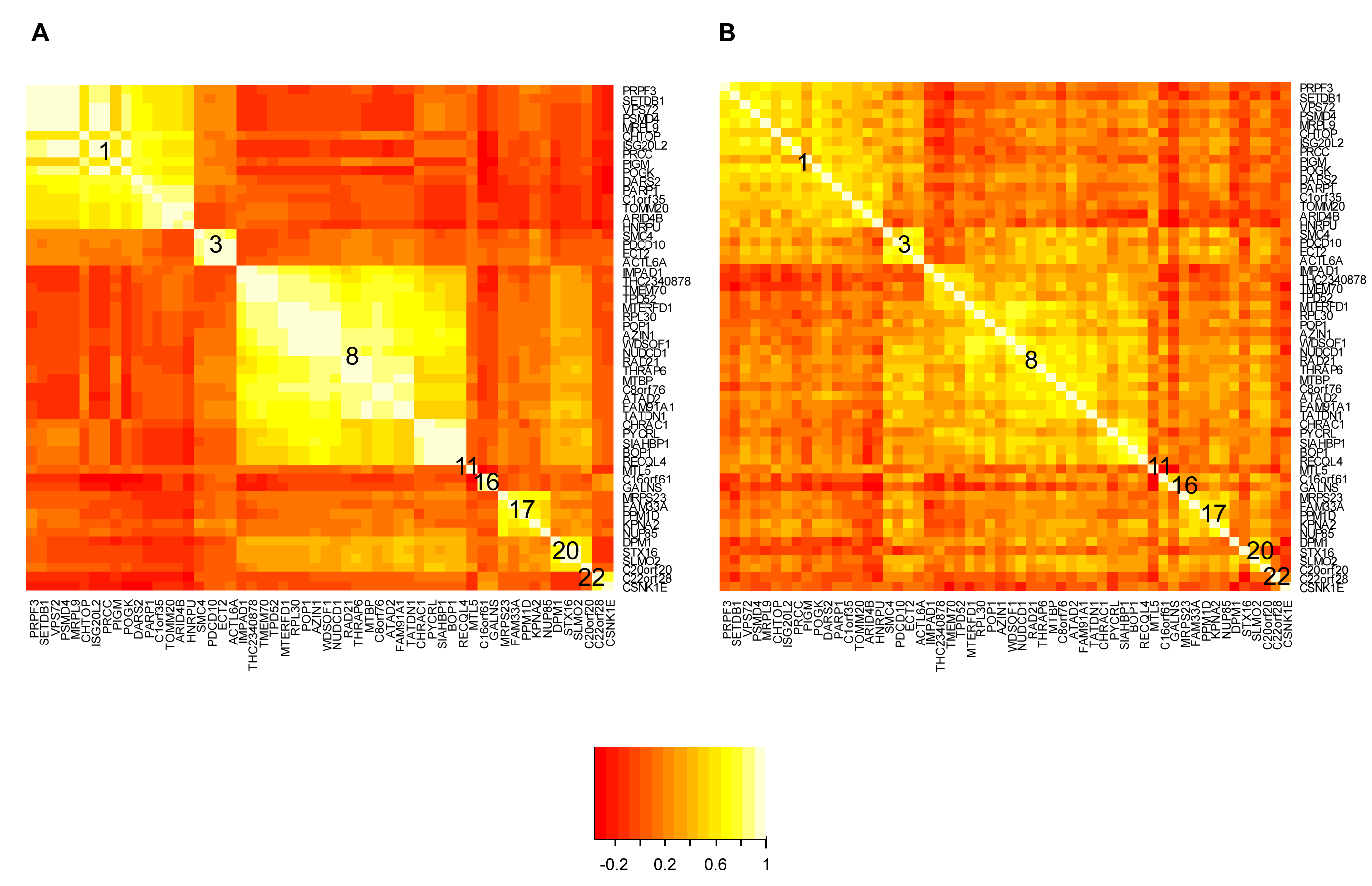

Supplement: Figure S7 — Correlation plots. (A) Pairwise correlations of log copy number of the 56 iPAC genes. (B) Pairwise correlations of log expression levels of the 56 iPAC genes. Chromosomes are indicated with numbers. (TIF) [file pone.0053014.s007.tif]

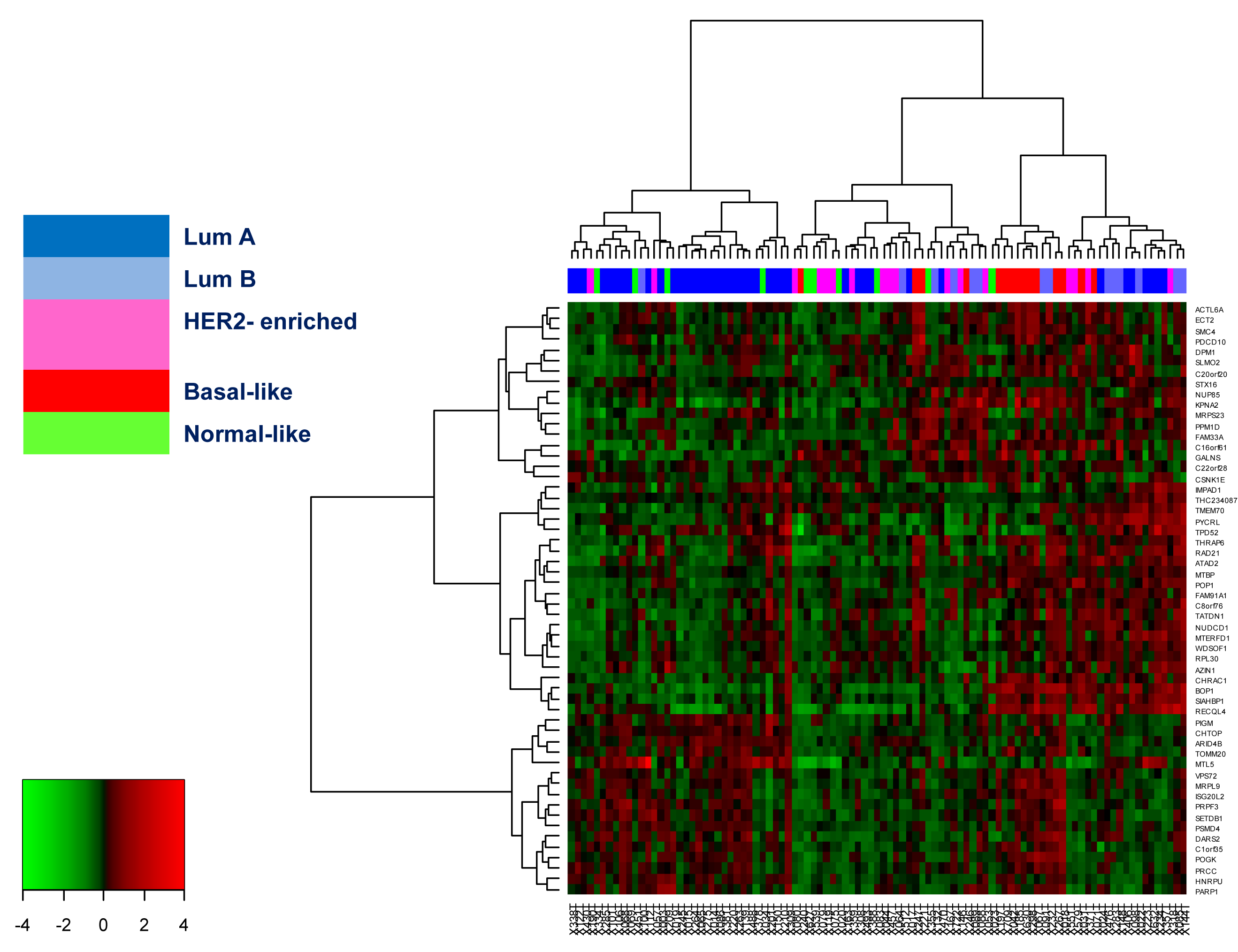

Supplement: Figure S8 — Hierarchical clustering of the expression levels of the 56 iPAC genes. Samples are color-coded according to gene expression subtype. The clustering was made with Pearson correlation using Ward linkage. Three samples could not be subtyped and were omitted from the analysis. Color map represents log expression values. (TIF) [file pone.0053014.s008.tif]

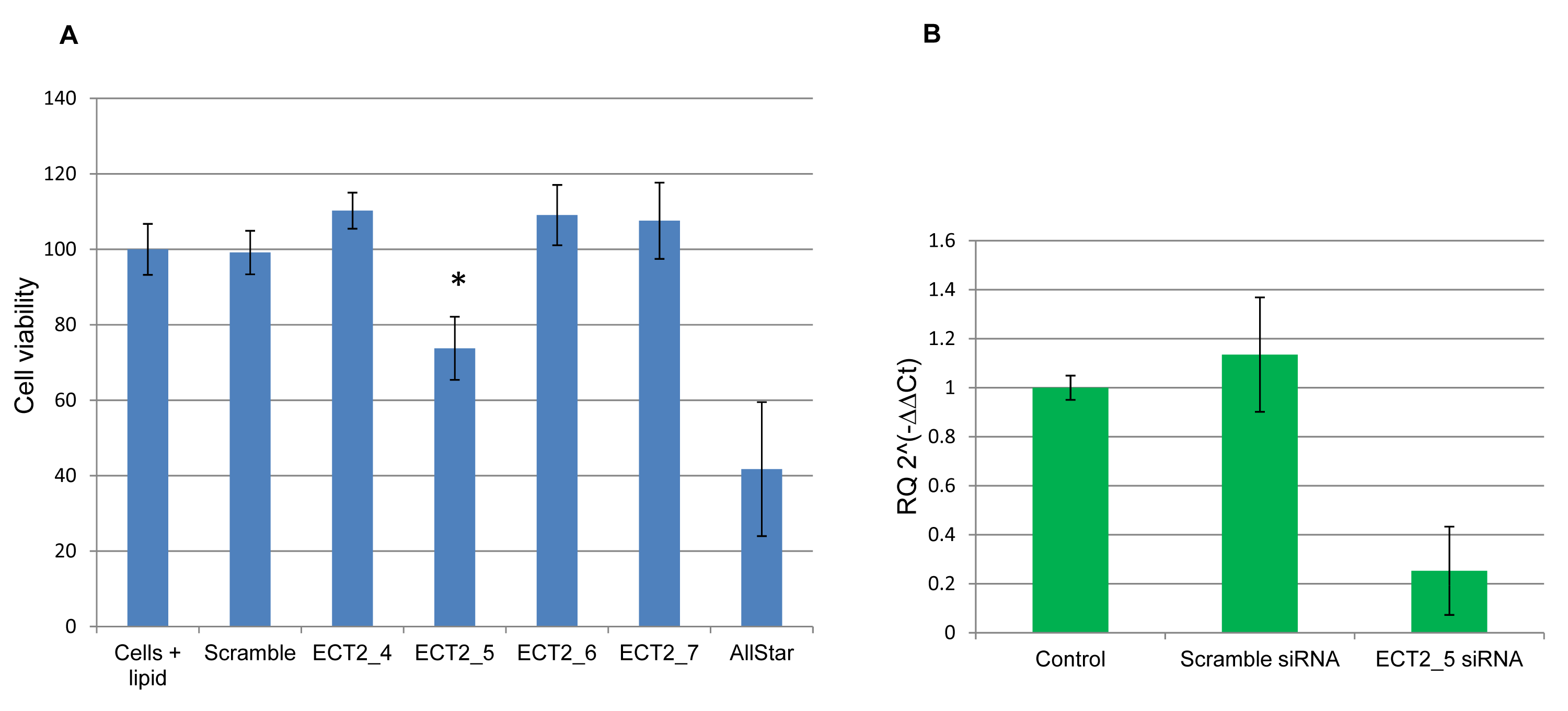

Supplement: Figure S9 — siRNA knockdown of the iPAC gene ECT2 . (A) Effect of siRNA knockdown of ECT2 on cell viability in the MCF7 cell line. Four various siRNAs against ECT2 were tested in addition to controls (bars show SD from eight replicates). The ECT2_5 siRNA shows a statistically significant reduction in cell viability compared to the non-transfected cells (asterisk; Student's t-test, p<0.05). (B) Relative quantification (RQ) of ECT2 mRNA after siRNA transfections (9 replicates), showing the specificity of the knockdown in the MCF7 cell line. The data were normalized to the control (cells + transfection lipid). (TIF) [file pone.0053014.s009.tif]
