# Supplementary material for: Identifying In-Trans Process Associated Genes in Breast Cancer by Integrated Analysis of Copy Number and Expression Data
Source: PLoS One. 2013 Jan 30;8(1):e53014. doi: 10.1371/journal.pone.0053014 (PMC3559658; doi:10.1371/journal.pone.0053014)
Supplement: File S1 — Cell culture and siRNA transfection. (PDF) [file pone.0053014.s014.pdf]

# Supporting Information

## Cell culture and siRNA transfection

MCF7 cells were obtained from Interlab Cell Line Collection (ICLC, Genova, Italy) and MDA-MB-231 from American Type Culture Collection (ATCC, Manassas, VA, USA). MCF7 cells were cultured in Dulbecco's Modified Eagle's Medium (DMEM, 1 g/l glucose, Sigma Aldrich, St. Louis, MO, USA) supplemented with 10 % fetal bovine serum (FBS, Gibco, Life Technologies, NY, USA), 2mM L-glutamine (Sigma Aldrich) and 1 % penicillin/streptomycin (P/S, Gibco). MDA-MB-231 were cultured in DMEM (4.5 g/l glucose) supplemented with 10 % FBS, 0.5 % P/S, 1x Glutamax (Gibco), and 20mM Hepes (Gibco).

Reverse transfection in 384 well plates (Greiner Bio-One GmbH, Frickenhausen, Germany) was conducted for three selected iPAC genes (*ECT2*, *MTBP*, and *PSMD4*), with four siRNAs targeting each gene. The siRNAs used were AllStars Negative Control siRNA (scrambled, negative control), AllStars Hs Cell Death Control siRNA (AllStar, positive control), Hs_ECT2_4, Hs_ECT2_5, Hs_ECT2_6, Hs_ECT2_7, Hs_MTBP_2, Hs_MTBP_5, Hs_MTBP_6, Hs_MTBP_7, Hs_PSMD4_5, Hs_PSMD4_6, Hs_PSMD4_10, and Hs_PSMD4_11 (Qiagen, Valencia, CA, USA). The four siRNAs for each gene were pooled together as well as all siRNAs as single siRNAs. Single siRNAs were used in 13 nM concentrations, and in 26 nM when pooled together. SiLentFect (BioRad, CA, USA) was used as transfection lipid. For both cell lines, the cell density was 1500 cells/well. The plates were incubated for 72h at 37°C with 5 % $CO_2$. CellTiter-Glo cell viability assay (Promega, WI, USA) using luminescence measurement with MicroBeta TriLux plate reader (PerkinElmer, MA, USA) was chosen as end-point. Both cell lines were validated with four technical and two biological replicates for *ECT2* and *PSMD4* (only four technical replicates for *MTBP*).

In order to validate that the Hs_ECT2_5 siRNA against *ECT2* was specific, relative quantification through real-time PCR of *ECT2* using TaqMan® Gene Expression Assay (Applied Biosystems, CA, USA) was performed. The transfection for this was performed in 24-well plates using 80 000 cells/well. The experiment was conducted with 3 technical replicates. After 72h incubation at 37°C with 5 % $CO_2$ the cells were collected and RNA isolated with TRIzol® (Invitrogen, CA, USA). *GAPDH* was used as an endogenous control. Reverse transcription PCR was carried out using the manufacturer's recommendation.

Quantitative Real-Time PCR was performed following the manufacturer's recommendation in triplicates on a 7900 HT Fast Real-Time PCR System (Applied Biosystems) with a standard absolute quantification thermal cycling program. Cycle threshold ($C_t$) values were obtained using the SDS 2.3 software (Applied Biosystems). An average $C_t$ value was calculated for each sample for both target and endogenous control, and the data were normalized by subtracting the average $C_t$ value of the endogenous control from the corresponding sample, thus obtaining the $\Delta C_t$ value. To make a relative quantification, the control samples were used as calibrator, and the samples normalized to the calibrator sample by subtracting the $\Delta C_t$ value of the calibrator, obtaining the $\Delta\Delta C_t$ value. Finally, $2^{-\Delta\Delta Ct}$ was used to represent the relative quantification.
